# Habitual Sleep, Sleep Duration Differential, and Weight Change Among Adults

**DOI:** 10.1093/geroni/igab046.129

**Published:** 2021-12-17

**Authors:** Yin Liu, Mari Palta, Jodi Barnet, Max Roberts, Erika Hagen, Paul Peppard, Eric Reither

**Affiliations:** 1 Utah State University, Logan, Utah, United States; 2 University of Wisconsin-Madison, Madison, Wisconsin, United States

## Abstract

We assessed longitudinal associations between diary-measured sleep duration and clinically assessed body mass index (BMI) among 784 men and women enrolled in the Wisconsin Sleep Cohort Study (mean [SD] age = 51.1 [8.0] years at baseline). The outcome was BMI (kg/m2). Key predictors were habitual sleep duration (defined as average weekday nighttime sleep duration) and sleep duration differential (defined as the difference between average weekday and average weekend nighttime sleep duration) at each data collection wave. Men with shorter habitual sleep duration on weekdays had higher BMI than men with longer habitual sleep duration on weekdays. Participants with larger differentials between weekday and weekend sleep duration experienced more rapid BMI gain over time for both men and women. Inadequate sleep, characterized as shorter habitual sleep during weekdays and larger weekday-weekend sleep differential, is positively associated with BMI levels and trajectories among men and women in mid-to-late life.

